# A Case of Acute Coronary Occlusion in a Morbidly Obese Patient Masked on Electrocardiogram by Low QRS Voltage

**DOI:** 10.1177/2324709620903133

**Published:** 2020-01-30

**Authors:** Jeffrey Golightly, Stacia Shipman, Ross Owens, Kelly Painter

**Affiliations:** 1 INTEGRIS Southwest Medical Center, Oklahoma City, OK, USA

**Keywords:** low QRS, morbid obesity, high BMI, masking EKG, acute coronary syndrome, ST-elevation myocardial infarction

## Abstract

Timely diagnosis of acute coronary occlusion is essential to avoid chronic cardiac impairment and death. We describe an uncommon case of acute myocardial infarction masked by low QRS voltage secondary to morbid obesity. This case highlights the importance of considering the degree of ST-segment elevation proportionally to the QRS amplitude if there is clinical suspicion of acute coronary occlusion.

## Introduction

Myocardial infarction (MI) is a major cause of morbidity and mortality worldwide. Timely diagnosis of an acute ST-segment elevation MI (STEMI) is important for reperfusion to prevent permanent myocardial damage. For this reason, the electrocardiogram (ECG) should be acquired and interpreted by a physician within 10 minutes after admission.^1^ The current criteria for STEMI requires the ST-segment elevation in leads V2-V3 to have J-point elevation ≥0.15 mV in women, ≥0.25 mV in men younger than 40 years, and ≥0.2 mV in men ≥40 years.^2^ In all other leads, an elevation of ≥0.1 mV is required. STEMI is diagnosed when a patient presents with persistent new ST-segment elevation in 2 or more anatomically contiguous leads in the context of a consistent clinical history.^3^ Low QRS voltage, defined as <1.0 mV in the precordial leads and <0.5 mV in the limb leads, has been found to be a poor prognostic indicator when present on initial ECG in the setting of acute coronary syndrome (ACS).^4,5^ Previous studies have shown that low QRS amplitude can be the result of processes that limit generation of voltage from the myocardium, such as MI or left ventricular hypertrophy.^6^ Low QRS voltage can also be seen when there is increased distance between the myocardium and the ECG leads, as with pericardial or pleural effusions or morbid obesity.^7^ There has been an ample amount of prior research showing that in the setting of ACS, the highest risk of mortality is found in low body mass index (BMI) patients.^8,9^ However, there has been no published research or case reports describing STEMI masked by low QRS voltage in high BMI patients.

In this article, we present a patient with diffuse low-voltage QRS amplitude with minimal ST-segment elevation, not meeting STEMI criteria, who had an acute coronary occlusion. We propose in similar cases that the degree of ST-segment deviation should be examined proportionally to the QRS amplitude if there is suspicion for acute MI.

## Case Presentation

A 66-year-old female with history of hypertension, hyperlipidemia, chronic myelocytic leukemia, and morbid obesity (BMI = 58 kg/m^2^) presented to the emergency department for chest pain. The pain woke her up from sleep just prior to arrival, and it was located in her left anterior chest, described as “sharp pressure” in nature. She denied any associated shortness of breath, nausea, or diaphoresis. Her vital signs were within normal limits. Physical examination revealed her to be in mild distress secondary to pain with trace pretibial edema bilaterally. She had normal lung sounds, and no cardiac murmurs were auscultated.

**Figure 1.**
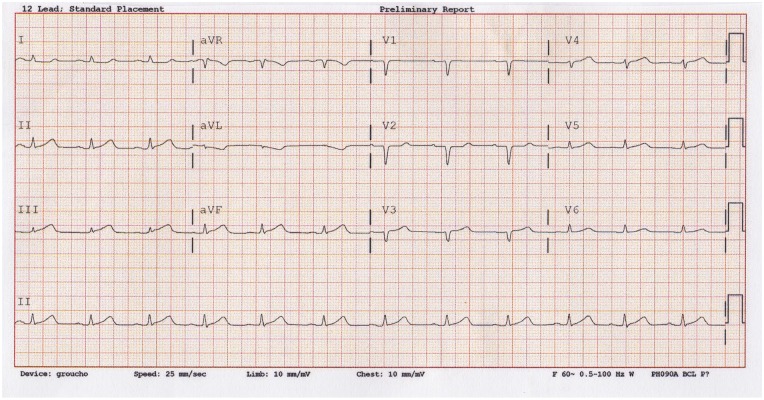
Initial ECG.

An initial ECG showed low QRS voltage with <1 mm ST elevation in lead III, slightly less ST elevation in aVF, and no ST elevation in II (Figure 1). Chest radiography showed no acute cardiopulmonary abnormalities. Laboratory markers revealed a minimally elevated troponin level of 0.045 ng/mL, otherwise normal basic metabolic panel and complete blood counts.

**Figure 2.**
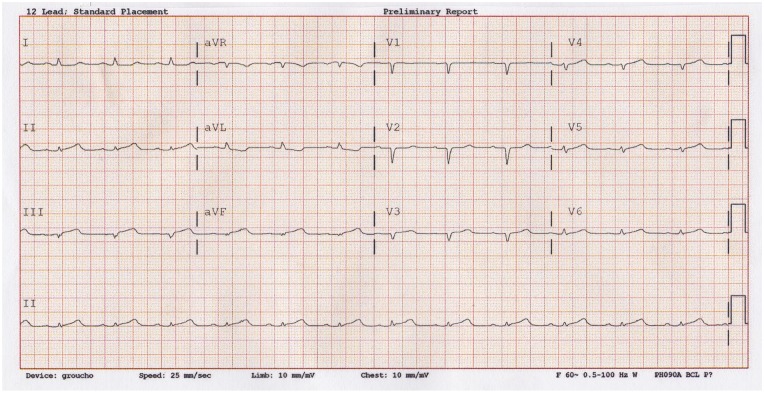
Repeat ECG.

The patient was given 324 mg of aspirin with improvement but not resolution of her symptoms. Repeat ECG was obtained 75 minutes later (Figure 2) showing low QRS voltage with worsening ST morphology and 1-mm ST elevation in lead III, slightly less ST elevation in aVF, and no ST elevation in lead II. Although the patient’s ECG still did not meet STEMI criteria (≥0.1 mV in 2 or more inferior leads), there was concern for an acute coronary occlusion given the patient had continued chest pain and ECG changes. There was also concern that the patient was actually having a STEMI, but that the proportion of the ST elevation was masked by the low QRS voltage in the setting of a high BMI patient.

Interventional cardiology was consulted, and it was decided to take the patient emergently to the cardiac catheterization laboratory for intervention. Coronary angiogram found a 99% thrombotic occlusion at the mid circumflex vessel successfully opened with a bare metal stent. The patient developed a short course of postoperative hypotension requiring dopamine infusion. She otherwise had an uneventful postoperative recovery and was discharged home in good condition 2 days later.

## Discussion

Low QRS voltage has been an indicator for adverse outcomes in the setting of ACS, and thought to indicate widespread nonviable myocardium for decades. A recent study found that patients with low QRS voltage had higher rates of multivessel coronary artery disease and that low voltage was an independent predictor for multivessel disease.8 High-frequency spectral analysis of changes in QRS amplitude and morphology during cardiac stress tests have been shown to be sensitive diagnostic markers of myocardial ischemia, often superior to measures of ST-T segment changes.^10^ Additionally, low QRS voltage on initial ECG in the setting of ACS has been found to be a poor prognostic indicator and is associated with higher incidence of prior MI, depressed left ventricular function, unadjusted 6-month mortality, in-hospital death, and myocardial reinfarction.^4,5^

The obesity paradox, described as a favorable prognosis in chronically ill patients with obesity, has been previously studied in patients with chronic kidney disease,^11^ chronic heart failure,^12^ and chronic obstructive pulmonary disease.^13^ More recently, a similar paradox linking higher BMI with better prognosis was described in coronary artery disease.^9,14,15^ A meta-analysis found the highest risk of mortality in the setting of ACS to be in low BMI patients, although the results should be interpreted with caution, as obese patients were younger and had less bleeding complications, which could have influenced survival.^9^

Obesity as a hindrance to timely diagnosis of ACS has not been previously studied or reported. To our knowledge, there has been no published research or case reports describing STEMI masked by low QRS voltage in morbidly obese patients. In our patient, we present a case of acute coronary occlusion whose ECG demonstrated diffuse low-voltage QRS amplitude with minimal ST-segment elevation, not meeting STEMI criteria. We suggest that in future cases of low QRS voltage, the degree of ST-segment elevation should be viewed proportionally to the QRS amplitude if there is clinical suspicion of acute coronary occlusion. This will aid providers to better evaluate cases of ACS that could benefit from emergent intervention and reperfusion to prevent permanent myocardial damage.
